# Interior Least Tern (*Sternula antillarum*) breeding distribution and ecology: implications for population-level studies and the evaluation of alternative management strategies on large, regulated rivers

**DOI:** 10.1002/ece3.726

**Published:** 2013-08-26

**Authors:** Casey A Lott, Robert L Wiley, Richard A Fischer, Paul D Hartfield, J Michael Scott

**Affiliations:** 1 American Bird ConservancyBoise, Idaho; 2 Consulting EcologistAthens, Ohio; 3 Envirnomental Laboratory, USACE Engineer Research and Development Centre (ERDC)Vicksburg, Mississippi; 4 Ecological Services, US Fish and Wildlife ServiceJackson, Mississippi; 5 Fish and Wildlife Sciences, University of IdahoMoscow, Idaho

**Keywords:** Dispersal, ecological response to dams and flow alteration, large river ecology, metapopulation management, population modeling, sandbar habitat, threatened and endangered species

## Abstract

Interior Least Terns (*Sternula antillarum*) (ILT) are colonial, fish-eating birds that breed within active channels of large sand bed rivers of the Great Plains and in the Lower Mississippi Valley. Multipurpose dams, irrigation structures, and engineered navigation systems have been present on these rivers for many decades. Despite severe alteration of channels and flow regimes, regulation era floods have remained effective at maintaining bare sandbar nesting habitat on many river segments and ILT populations have been stable or expanding since they were listed as endangered in 1985. We used ILT breeding colony locations from 2002 to 2012 and dispersal information to identify 16 populations and 48 subpopulations. More than 90% of ILT and >83% of river km with suitable nesting habitat occur within the two largest populations. However, replicate populations remain throughout the entire historical, geophysical, and ecological range of ILT. Rapid colonization of anthropogenic habitats in areas that were not historically occupied suggests metapopulation dynamics. The highest likelihood of demographic connectivity among ILT populations occurs across the Southern Plains and the Lower Mississippi River, which may be demographically connected with Least Tern populations on the Gulf Coast. Paired ecological and bird population models are needed to test whether previously articulated threats limit ILT population growth and to determine if management intervention is necessary and where. Given current knowledge, the largest sources of model uncertainty will be: (1) uncertainty in relationships between high flow events and subsequent sandbar characteristics and (2) uncertainty regarding the frequency of dispersal among population subunits. We recommend research strategies to reduce these uncertainties.

## Introduction

**Figure 1:**
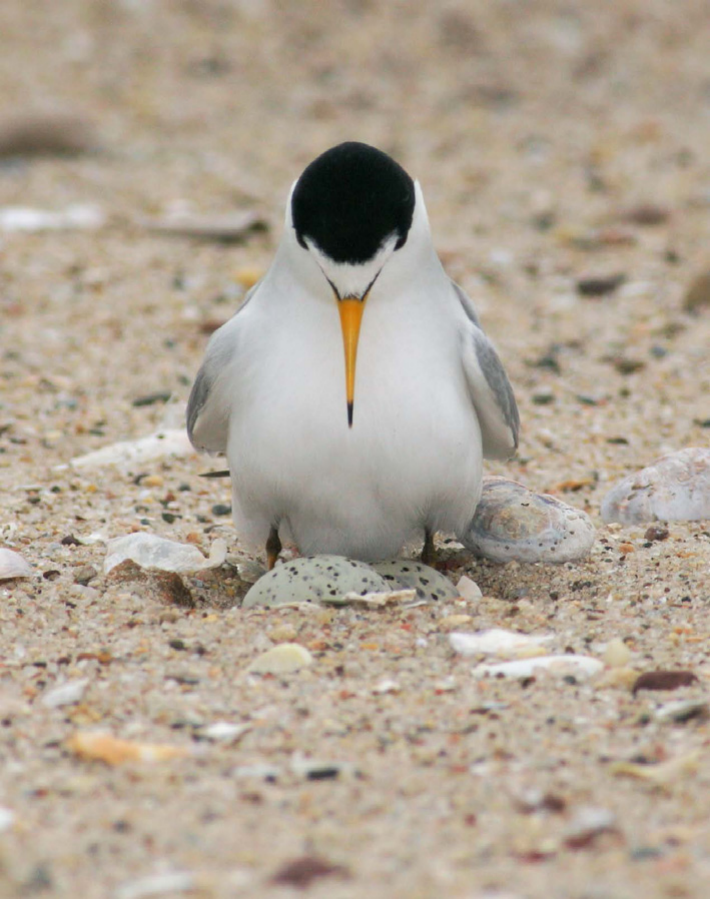
“Interior” Least Tern (*Sternula antillarum*) nesting on a large river sandbar. Photo: Tom Grey.

**Figure 2:**
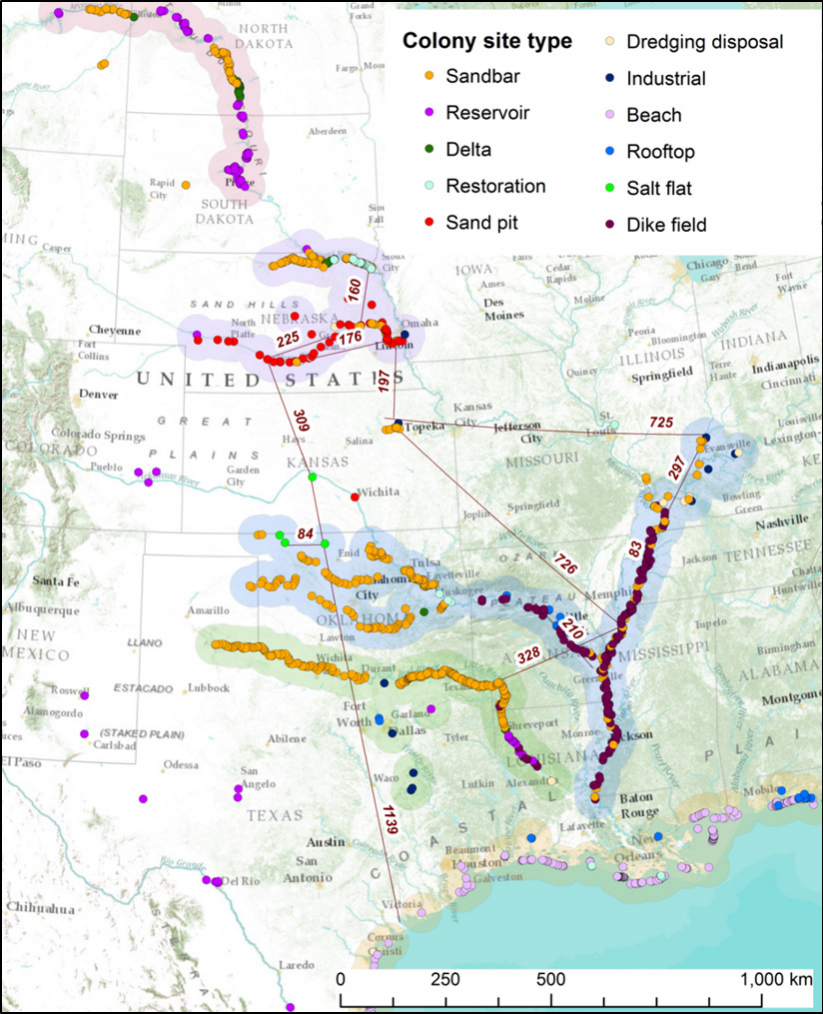
This map displays the 4 major ILT “populations” identified in our GIS analysis: Blue shading, the Lower Mississippi, Arkansas, Canadian, and Cimarron rivers; Green, the Red and Trinity Rivers; Purple, the Platte, Niobrara, and Missouri Rivers (below Fort Randall and Gavins Pt. dams); Pink, Northern Missouri River. The Gulf Coast Least Tern population is shown (beige shading) for spatial reference. Brown lines labeled by brown numbers illustrate 13 ILT band recoveries reflecting dispersal distances >80 km (unpublished data from the U.S. Geological Survey, Patuxent Wildlife Research Center, Bird Banding Laboratory). The true frequency of dispersal events >80 km, which would connect many ILT populations and some ILT populations with Gulf Coast populations, is unknown due to the absence of unbiased dispersal information for this species.

Least terns (*Sternula antillarum*) are colonial, fish-eating birds that nest on barren sandy substrates in open habitats on rivers and coasts (Thompson et al. 1997) (Fig. 1). The US Fish and Wildlife Service (USFWS) listed “interior” populations of Least Terns (ILT) as endangered due to perceived low numbers and concerns about breeding habitat loss associated with water resource projects on large rivers in the central US (USFWS 1985, 1990). The USFWS defined the ILT population as all Least Terns nesting on or adjacent to the major rivers of the Great Plains and the Lower Mississippi Valley (north of Baton Rouge, LA), and along Texas rivers >80 km from the Gulf of Mexico coast (USFWS 1985). ILT breeding populations are distributed from the Missouri River in Montana south through the Mississippi River Valley and its large western tributaries, with large distributional gaps along this river network due to the presence of multiple reservoirs and the channelized Lower Missouri River Navigation System (Funk and Robison 1974; Johnson 2002; USFWS 2005a; Lott 2006) (Fig. 2, Table 1).

**Table 1:** Suggested population and subpopulation subunits for studies of ILT population dynamics (numbers match Figs. 2 and 4–6)

Pop.	Population/subpopulation	River Km.	*N* sites	Med. ICD	Min. Count	Max. Count	Max% ILT
1.1	Ft. Peck Lake^1^	−	1	−	0	2	0.0
1.2	Missouri R. bars bel. Ft. Peck Dam^1^	182	4	13	22	77	0.4
1.3	Lake Sakakawea^1^	−	3	11	11	53	0.3
1.4	Missouri R. bars bel. Garrison Dam and Lake Oahe^1^	148	16	22	179	309	1.8
1	*Northern Upper Missouri R*.	330	24		217	439	2.5
2	Yellowstone R.^2^	−	2	9	14	19	0.1
3	Cheyenne R.^3^	−	1	−	3	8	0.0
4.1	Niobrara R bars., Missouri R. bars bel. Ft. Randall and Gavins Pt. Dams and Lewis and Clark Lake^1,4,5^	287	40	6	569	845	4.8
4.2	Lake McConaughy and west. Platte R. sand pits^6^	−	4	38	52	−	−
4.3	Lower Platte R. bars and sand pits and Central Platte and Loup R. sand pits^7,8,9^	156	49	5	642	−	−
4.4	North Loup R. sand pits- A^7^	−	1	−	4	−	−
4.5	North Loup River sand pits- B^7^	−	1	−	10	−	−
4.6	Elkhorn R. sand pits^7^	−	2	33	74	−	−
4	*Niobrara, Platte, and So. Upper Missouri R*.	443	97		1627	−	−
5	Kansas R. bars and industrial^10^	43	5	11	34	45	0.3
6	Arkansas R. reservoirs in Colorado^11^	−	3	33	38	50	0.3
7	Quivira NWR salt flats^12^	−	1	−	17	40	0.2
8	Wichita sand pit near Arkansas R.^13^	−	1	−	8	18	0.1
9	Mississippi R. barge site near St. Louis^14^	−	1	−	24	42	0.2
10.1	Western Cimarron R. bars^12^	10	2	9	12	12	0.1
10.2	Cimarron R. salt flats^12^	−	2	22	191	242	1.4
10.3	Salt Plains NWR salt flats^15^	−	1	−	90	263	1.5
10.4	Cimarron R. bars^12^	256	21	9	176	176	1.0
10.5	Western Canadian R. bars^12^	160	16	7	58	58	0.3
10.6	Canadian R. bars^12^	353	31	7	280	280	1.6
10.7	Canadian R. delta at Eufaula Lake^12^	−	1	−	130	−	−
10.8	Arkansas R. bars below Kaw Dam^16^	119	13	5	93	201	1.1
10.9	Arkansas R. bars bel. Keystone Dam, Canadian R. bars bel. Eufaula Dam, and restor. sites Kerr Lake^16^	131	35	3	437	699	4.0
10.10	Ark. Nav. System dike fields: Above Ozark Lake^17,18^	10	2	4	8	13	0.1
10.11	Ark. Nav. Sys. dike fields: Above Dardanelle^17,18^	13	3	8	31	66	0.4
10.12	Ark. Nav. Sys.dike fields: Dardanelle- Little Rock^17,18^	69	10	6	96	104	0.6
10.13	Low. Mississippi R. dike fields, Low. Arkansas R. bars, Ark. Nav. Sys. dike fields bel. Little Rock and Low. Ohio R.^17-20^	1283	79	9	8295	13,135	74.7
10.14	Wabash R. bars, Gibson Lake indust. and restor.^21^	107	4	9	107	280	1.6
10.15	Near Ohio and Wabash R. confluence^22^	−	2	24	27	27	0.2
10.16	Ohio R. dredging site and AEP industrial site^22^	−	2	8	36	36	0.2
10.17	Ohio R. bars, disposal sites and Arkema indust.^22^	−	4	8	100	100	0.6
10.18	Middle Mississippi R. upstream of Cairo, IL^37^	8	3	23	12	52	0.3
10	*Mississippi, Arkansas, Canadian, and Cimarron R*.^10-22^	2562	231		13,360	−	−
11.1	Upper Red R. bars^12^	507	59	6	394	597	3.4
11.2	Hagerman NWR indust. Site^23^	−	1	−	p	p	−
11.3	Red R. bars bel. Denison Dam^24-26^	761	79	5	1070	1376	7.8
11.4	Red River Nav. Sys. disposal site^26^	−	1	−	18	51	0.3
11.5	Cooper Lake^27^	−	1	−	45	90	0.5
11.6	Dallas Area rooftops and industrial sites^28^	−	3	25	63	114	0.6
11.7	Trinity R. mines^29^	−	3	21	44	88	0.5
11	*Red and Trinity R*.^12,23-29^	1268	147		2008	−	−
12.1	Bitter Lake Reservoir on Pecos R.^30^	−	1	−	22	28	0.2
12.2	Brantley Lake Reservoir on Pecos R.^31^	−	1	−	11	−	−
13	Imperial Reservoir on Pecos R.^23^	−	1	−	14	−	−
14	Twin Buttes and O.C. Green Reservoirs, Conchos R.^23^	−	2	20	p	p	−
15	Amistad Reservoir on Rio Grande R.^32^	−	4	7	85	278	1.6
16	Falcon Reservoir on Rio Grande R.^23^	−	1	−	p	p	−
ALL	*All Interior Least Tern Populations*^1-32^	4603	523	−	17,591	18,000	−
	Four *main Interior Least Tern Populations (1,4,10,11)*^*1,4-30*^	4603	499	−	17,212	−	−
17	*Gulf of Mexico coast*^33-36^		348	−	11,400	12,200	

Superscripts reference data sources listed in acknowledgments. River km, the continuous linear extent of river along which ILT colonies occurred within ≤24 km of each other from 2002 to 2011; *N* sites, the number of nesting sites within each population/subpopulation; Median ICD, median intercolony distance (in km) for all colonies within a subpopulation; Min. and max. of annual counts from 2002 to 2011. Max% ILT, max. count between 2002 and 2011 divided by 17,591, the number of adult ILT counted during the only range-wide survey for this population (Lott 2006).

Decades of research and the current conservation status of ILT are based on the assumption that historical engineering on large rivers would impact negatively the breeding biology and population growth of ILT (USFWS 1985, 1990; Guilfoyle and Fischer 2012). This is based on the simple and reasonable idea that the filling of reservoirs behind large dams and altered flow regimes on regulated rivers might decrease the amount or quality of ILT nesting sandbars. In this paper, we propose a formal definition of ILT habitat and describe the current distribution of ILT based on colony locations reported during the 10 year period of 2002–2011. We then map colony locations as populations and subpopulations based on ILT dispersal distances and frequencies. We describe the characteristics of each of these populations (e.g., numbers of colony sites, distances among colony sites, and ILT counts) relative to regulation era ecological processes that continue to form and maintain suitable nesting habitat across the range of ILT. Given this baseline, we discuss how the fragmented distribution of ILT on regulated rivers may affect ILT population dynamics, describe important sources of uncertainty in drawing inferences about ILT population process, and suggest future research to help reduce these uncertainties.

### The effects of river engineering on the distribution of ILT sandbar nesting habitat

Dam placement and the construction of navigation systems resulted in an initial pulse of ILT nesting habitat loss, followed by more gradual habitat loss and degradation as channels downstream of dams adjusted to altered flow regimes (Williams and Wolman 1984; Johnson 1997; Friedman et al. 1998). Much of this initial habitat loss and degradation will be permanent without dam removal or drastic changes to water management policies that have been institutionalized, and tested in court, for decades (USFWS 2003; USDOI 2006). The current distribution of ILT is a patchwork of populations distributed across dike-field sand deposits within navigation systems (USACE 1999, 2005; USFWS 2005b); relict river segments, *sensu* Johnson (2002), below large multipurpose dams (USFWS 2003, 2005a; Lott 2006); and anthropogenic sites along altered river channels (Sidle and Kirsch 1993) (Fig. 2, Table 1). Consequently, ILT population persistence is strongly influenced by: (1) continued construction and maintenance of dikes and dredged material management within navigation systems (Smith et al. 1988; USFWS 2012); (2) the persistence of ecological processes that maintain sandbar nesting habitat on discrete river segments above and below large dams (Sidle et al. 1993; Leslie et al. 2000; USACE 2011; Lott and Wiley 2012a,b); and (3) the degree to which fragmented breeding populations are isolated demographically; which depends on dispersal frequency among population subunits (Hanski 2002; Akcakaya et al. 2003; Reed and Levine 2005).

Previous descriptions of ILT distribution have emphasized administrative rather than ecological definitions of breeding populations (USFWS 1985, 1990, 2003, 2005a). We find this problematic when the primary concerns regarding ILT habitat response to river engineering have ecological underpinnings. In this paper, we consolidate data from multiple sources to redefine ILT populations relative to the major biological (e.g., dispersal, habitat selection) and ecological (e.g., vegetation succession relative to flood frequency) factors that constrain their distribution. We define discrete populations and subpopulations based on habitat connectivity and the decreasing likelihood of dispersal among occupied habitat patches at greater distances (Akcakaya et al. 2003). This approach provides a standard set of distributional subunits for the study of ILT population dynamics.

### A biological definition of sandbar nesting habitat for ILT

Lott and Wiley (2012a) proposed a resource-based definition of ILT “sandbar nesting habitat” as the physical and biological resources necessary to sustain ILT reproduction. This definition focuses habitat description on ILT life history and nesting behavior. We prefer this definition to the widely used term “emergent sandbar habitat,” which refers to the land-cover type of bare sand, often delineated from aerial photography (USACE 1999; USFWS 2003; Sherfy et al. 2008). Four main factors determine the subset of bare sand areas that ILT use for nesting: (1) sandbar elevation; (2) distance from large shrubs and trees (>2 m high); (3) the absence or paucity of sandbar vegetation; and (4) the availability of small fishes within 10 km of areas that meet criteria 1–3.

Sandbar elevation is the primary determinant of nesting habitat availability and quality (Ziewitz et al. 1992; USACE 2011; Lott and Wiley 2012a). Nesting sandbars must remain continuously exposed for the entirety of the egg laying, incubation, and flightless chick-rearing periods for successful ILT reproduction (Smith and Renken 1991; Lott and Wiley 2012a). Consequently, low elevation portions of sandbars that may appear as bare sand during low river stages and are regularly inundated during the breeding season and are not suitable nesting habitat (USACE 2011; Lott and Wiley 2012a).

On the Missouri River, ILT rarely nest within 150 m of shrubs or trees (>2 m in height) or other features (e.g., bluffs, bridges, or power lines) that provide high perches for avian predators or forest patches that may support mammalian predator communities (USACE 2011). Similarly, on the Lower Platte River between 1999 and 2008, all Least Tern nests were located on sandbars where channels were at least 300 m wide (Jorgensen et al. 2012). Least Terns are absent from sandy rivers with narrow channels and forested banks, even when bare sand is seasonally available for nesting and forage fish are abundant (e.g., the Republican and Elkhorn Rivers in Nebraska). In some cases, Least Tern populations may occur on narrow channels (e.g., 250 m wide) when farming directly to the river's bank has resulted in the removal of large trees (USACE 2011) or when trees have been purposefully removed for nesting habitat restoration (Plettner and Jenniges 1999). On sand pits associated with sand and gravel mining, some ILT nest closer to ≥2 m tall vegetation than on rivers. For example, of 149 nests on Central Platte River sand pits in 2010 and 2011, 37 were <133 m from the nearest tall vegetation (David Baasch, Platte River Recovery Program, unpubl. data). This information suggests that a small number of ILT may nest closer than 150 m from large trees. However, within river channels, distances greater than 150 m are much more common and may be preferable (USACE 2011). The vast majority of nests for which measurements are available have been placed at least this far from shrubs or trees (Baasch 2011; USACE 2011; Jorgensen et al. 2012).

Least Terns also avoid portions of sandbars that are covered in low vegetation (Thompson et al. 1997; USACE 2011; Sherfy et al. 2012b). During periods with few floods, vegetation succession can render nesting habitat unsuitable for this reason, as Least Terns will abandon colony sites with abundant low vegetation cover (Burger 1984). During drought periods when large floods are infrequent, plant succession may advance to late seral stages, resulting in temporary changes in the distribution of regional breeding sites (USACE 2011); or, in extreme cases, permanent habitat loss due to channel narrowing via tree recruitment within river channels (Stinnett et al. 1988; Ziewitz et al. 1992).

ILT eat a variety of small, narrow-bodied, surface-swimming fish species (Thompson et al. 1997). For this reason, nesting habitat must be located within foraging range of water bodies that provide small fishes to support adult survival and chick growth. A recent radio-telemetry study documented foraging distances of up to 10 km from nesting colonies (Sherfy et al. 2012a). In some locations, the unavailability of fish across the entire breeding season may preclude ILT nesting in areas that are suitable in all other ways. For example, wide, sandy channels with high-elevation sandbars are present on upper portions of the Red, Canadian, and Cimarron rivers in southwestern Great Plains areas with low precipitation (Durham and Wilde 2006). Some of these areas may lack ILT due to falling late-summer hydrographs that dry out channels and reduce fish populations (Falke et al. 2011).

## Methods

**Figure 3:**
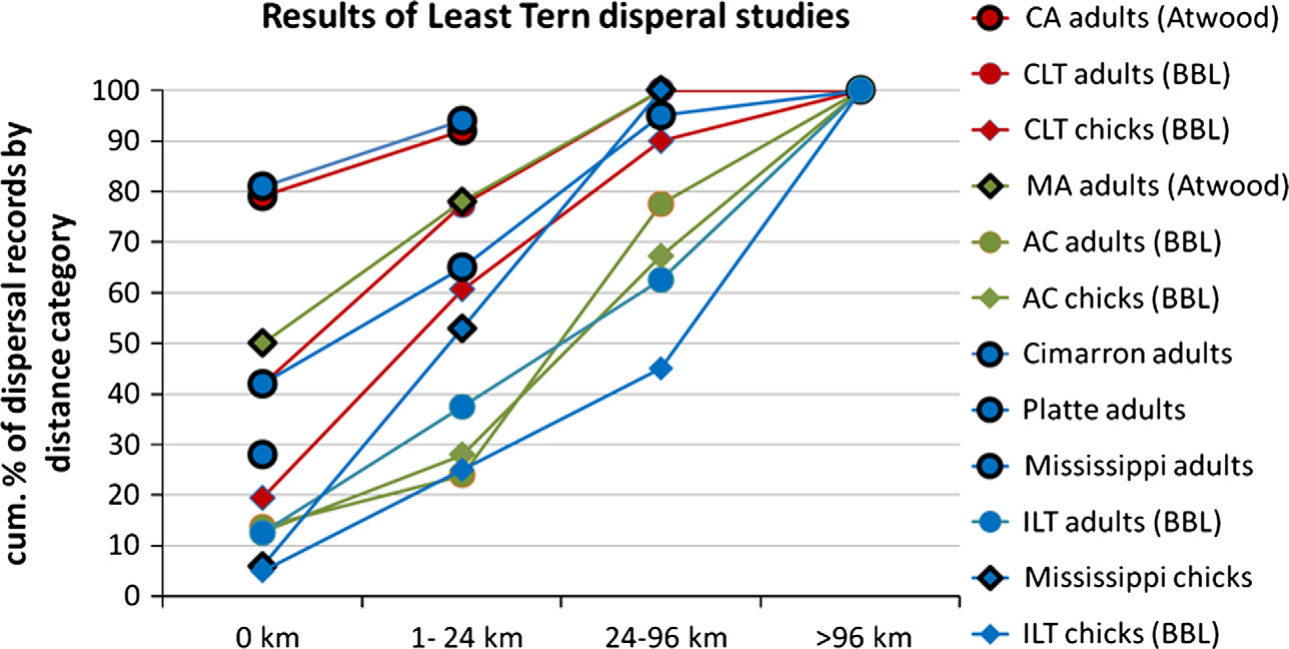
Dispersal distance frequencies, by age class, from published literature (Atwood and Massey 1988, Boyd 1993, Lingle 1993; Renken and Smith 1995b; Akcakaya et al. 2003) and 1261 band recovery records from 1923 through 2011, obtained from the U.S. Geological Survey, Patuxent Wildlife Research Center, Bird Banding Laboratory. Published studies had variably sized study areas, all of which were restricted to <96 km from the original banding site. These studies would only produce long-distance band recoveries in the unlikely event that banded individuals were found dead outside of these small study areas or captured again by researchers in distant locations.

To define discrete ILT breeding populations, we reviewed >200 published papers and unpublished reports on ILT and submitted information requests to >50 biologists working on rivers with ILT. Our objective was to acquire all ILT colony locations documented in the 10 years between 2002 and 2011. We compiled this information into a Geographic Information System (GIS) for additional analyses (ESRI 2010). Following Garton (2002), we define a breeding “population” as a collection of nest and/or colony locations that are “connected through frequent dispersal, occupying a collection of habitat patches that lack large intervening areas of nonhabitat relative to dispersal distances.” To group colony locations into discrete breeding populations, we compiled dispersal distance frequencies by age class from all available sources (Fig. 3). Due to uncertainty regarding Least Tern dispersal distances, we also defined “subpopulations” within populations (see below).

We defined ILT populations and subpopulations in ArcGIS 9.1 (ESRI 2010) following Akcakaya et al. (2003), by plotting colony locations from 2002 to 2011, buffering colonies by common dispersal distances (Fig. 3), and then dissolving buffers to create continuous polygons representing populations and subpopulations within which dispersal would be more likely than dispersal among polygons. We used a 24 km colony buffer (e.g., a 12 km radius from colony locations) to define subpopulations (given the high frequency of adult site fidelity, natal site philopatry, and short distance dispersal events) and a 96 km buffer to define populations, given the moderate frequency of dispersal distances between 25 and 96 km and the low frequency of observed dispersal distances >96 km (see citations in Fig. 3).

We summarized the following characteristics for each of the populations and subpopulations identified in the analysis above: (1) the number of colony sites within each subpopulation (from a randomly chosen year among those with data available between 2002 and 2011); (2) the continuous linear extent (in km along river centerlines) of occupied habitat; (3) the median intercolony distance (ICD, in km) for all pairs of colonies within a subpopulation, arranged linearly along the channel; and (4) the maximum count between 2002 and 2011 as a percentage of the range-wide count of 17,571 adult ILT in 2005 (Lott 2006) (Table 1). We view these percentages as coarse estimates, as 2005 survey data came from multiple sources, none of which employed protocols to estimate detection probabilities, which are known to vary with observer, sampling methods, and environmental conditions (Lott 2006; Nichols et al. 2009). ICD analyses were done with Hawth's tools in ArcGIS 9.1 (ESRI 2010) using colony locations from a single breeding season only (the exact year was selected randomly from the variable subset of years for which we had data for different areas). Figures 5 display colony locations by 1 of 11 different site types (e.g., sandbar, dike field, etc.), which we assigned from notes in reports or publications and/or by viewing colony locations on aerial photography. Our delineation of “populations” and “subpopulations” used data only on colony locations and potential dispersal distances. Consequently, several of our subpopulations comprised multiple contiguous river segments, with different channel characteristics and flow regimes. For example, subpopulation 4.1 includes the Niobrara River, relict riverine segments below Fort Randall and Gavins Point Dams on the Missouri River, and the sediment delta of the Niobrara River where it enters the Missouri River at Lewis and Clark Lake (Fig. 5).

**Figure 4:**
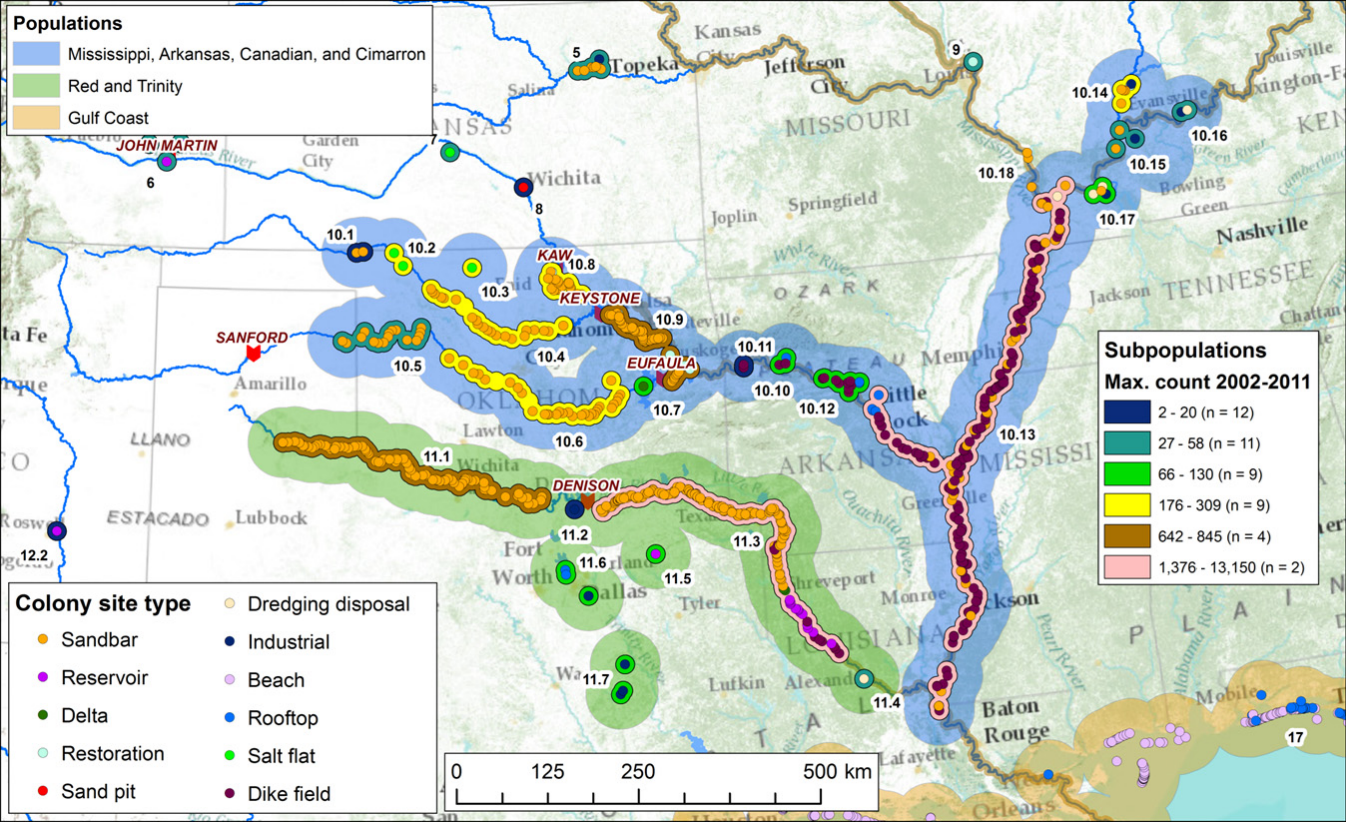
Detailed map of ILT populations 5–11 (and their constituent numbered subpopulations) spanning the Southern Great Plains and the Lower Mississippi River valley. See Table 1 for more information. All-caps text above red chevrons gives major dam names.

**Figure 5:**
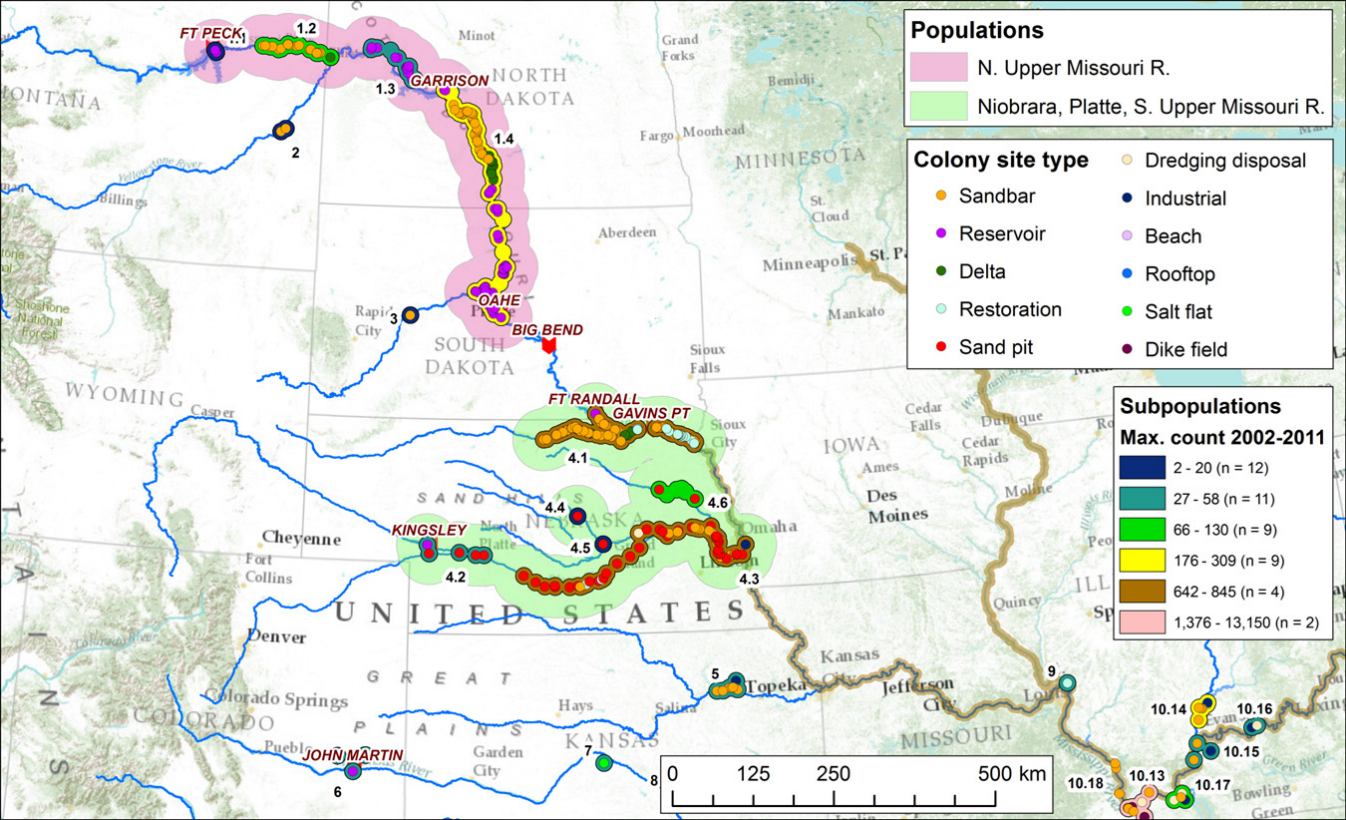
Detailed map of ILT populations 1-4 (and their constituent numbered subpopulations) spanning the Northern Great Plains. See Table 1 for more information. All-caps text above red chevrons gives major dam names.

## Results

**Figure 6:**
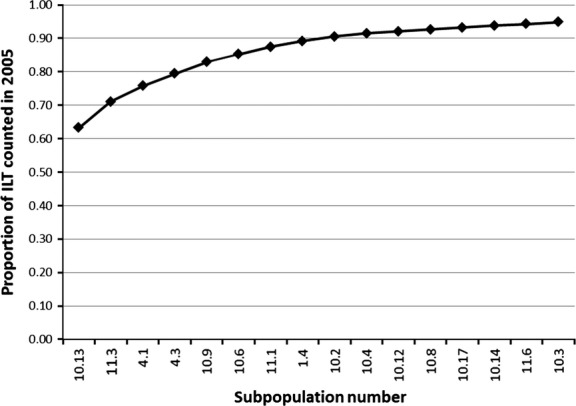
The cumulative contribution of the top 16 (out of 48) subpopulations to the total ILT population based on counts from 2005, the only year for which count data are available for all populations (Lott 2006). Note that only 9 of 48 subpopulations comprised ∼90% of all ILT and that 16 subpopulations comprised 95%. Each of these 16 subpopulations had counts >90 adults in 2005. Subpopulation numbers match Figures 4 and 5 and Table 1.

We defined 16 discrete “populations” and 48 “subpopulations” across the range of ILT (Figs. 2, 4, and 5; Table 1). Twelve small populations had annual counts <50 adults and <4 colonies. Collectively, these minor populations comprise <5% of all ILT (Table 1) and we do not discuss these further, except to note that many of these populations are present along the periphery of the ILT range and associated with anthropogenic habitat types (Figs. 5). Figure 6 illustrates the cumulative contribution of the top 16 (of 48) subpopulations to the total ILT population based on counts from 2005, the only year for which count data are available for all populations (Lott 2006).

Greater than 90% of all ILT and 83% of the 4600 linear river km with suitable nesting habitat occur within two large populations in the southern portion of the ILT range (populations 10 and 11, Figs. 2 and 4). Population 10, which contained 76% of all ILT during the range-wide survey of 2005, includes the dike fields of the Lower Mississippi River Navigation System; dredged material islands of the Ohio River Navigation System; sandbars, industrial, and restoration sites on the Wabash River; dike-field sandbars, restoration sandbars, relict river segments below dams on the Arkansas River; and above-dam river segments on the Cimarron and Canadian Rivers. Population 11 includes sandbar nesting areas above and below Denison Dam on the Red River and industrial sites along the Trinity River. Many subpopulations within populations 10 and 11 are <120 km of each other and several are <200 km from the Gulf of Mexico Coast (Fig. 4). If we assume dispersal is most likely between nearby colonies, as is the case for Least Terns on the Atlantic Coast (Akcakaya et al. 2003), the highest likelihood of demographic connectivity among ILT subpopulations is across the Southern Plains and the Lower Mississippi River, which may also be demographically connected with Least Tern breeding populations on the Gulf of Mexico coast (Boyd and Thompson 1985; Kirsch and Sidle 1999; Lott 2006) (Fig. 4).

Two small populations in the northern portion of the ILT range account for 5% of all ILT (Fig. 2 and 5, Table 1). Population 4 includes the Niobrara River, two relict reaches of the Missouri River below dams, Elkhorn River sand pits, Loup River sandbars and sand pits, and Platte River sandbars and sand pits. Colonies in population 4 are >400 km from colonies in the Southern Plains and >700 km from the closest colonies on the Lower Mississippi River, with its extremely high density of terns. Population 1, within the far northern portion of the ILT range, contains <1% of all ILT (Table 1). It includes two relict river segments below Missouri River dams and three small populations along the shorelines of Missouri River reservoirs (Fig. 5, Table 1). Colonies in population 1 are >200 km from the closest colonies in population 4. Again, if we assume that dispersal is most likely between nearby colonies, population 4 (and especially population 1) has a higher likelihood of demographic isolation from the rest of the ILT range than even the likelihood that ILT populations 10 and 11 are isolated from Least Tern populations on the Gulf Coast.

## Discussion

**Figure 7:**
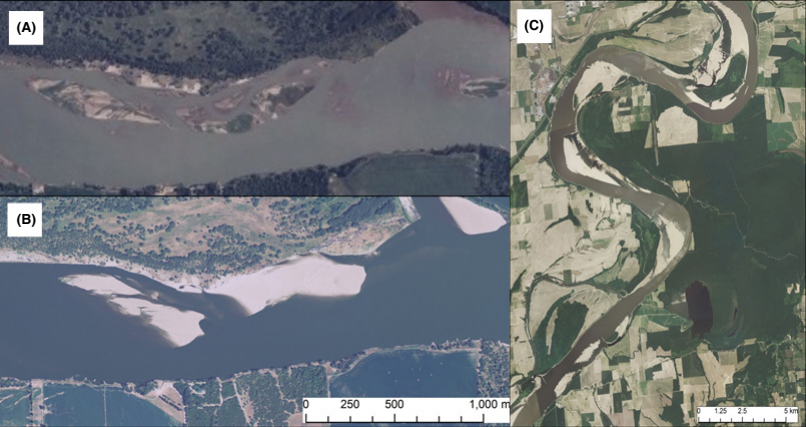
Images A and B show the same bend of the Missouri River below Gavins Pt. Dam. Pioneering vegetation on sandbars in image A (taken in 2005) reflects the absence of high flows in the 8 years since high dam releases in 1997. Image B (taken in 2011) shows large, bare sandbars in the same location after extended high dam releases in 2011. These paired images illustrate the pattern of sandbar nesting habitat renewal for ILT on regulated rivers with periodically high flood control releases. Image C illustrates dike-field sand accumulations on the Lower Mississippi River (LMR) navigation system, which provide the most extensive bare sand nesting habitat across the geographic range of ILT. Dike-fields on inside bends trap sediment to provide deep, sediment-free channels on outside bends for barge navigation. Intra-annual stage differences that routinely exceed 30 feet keep most of these sand accumulations free of vegetation. Note the much larger size of dike field sandbars on the Mississippi (3-5 km in length, see scale bar) than on the Missouri (sandbars less than a half-km in length).

Landscape structure and dispersal behavior are critical to understanding population dynamics (Hanski 1999; Reed and Levine 2005). Extensive river alteration in the central US has caused habitat fragmentation for species that strongly associate with large rivers, including ILT (USFWS 2003, 2005a; Perkin and Gido 2011; Wildhaber et al. 2011). Despite this fragmentation, the distribution of ILT has been stable to expanding over the past 50 years: see Figure 2 in (Hardy 1957); Figure 7 in (Ducey 1981); Figure 1 in (Kirsch and Sidle 1999); Figure 1 in (Lott 2006); and Figures 2, 4, and 5, this publication. The degree to which habitat fragmentation decreases population viability depends on landscape structure (e.g., species with more fragmented distributions are more susceptible to extinction) and the ability of species to disperse among fragmented habitat patches (e.g., fragmentation is less problematic for species with long-distance dispersal capabilities) (Hanski 1999). Garton (2002) defined a “metapopulation” as “a collection of populations sufficiently close together that dispersing individuals from source populations readily colonize empty habitat patches…”. Colonization of anthropogenic habitats by ILT (reservoirs, rooftops, mines, industrial sites, etc.), particularly in areas not known to have been historically occupied by the species, suggests metapopulation dynamics.

For this publication, we aggregated ILT colony locations into “subpopulation” and “population” subunits based on dispersal distances from empirical studies (Fig. 3). These studies have documented a small number of long-distance dispersal events (Boyd and Thompson 1985; Renken and Smith 1995a; Boyd and Sexson 2004) (Figs. 3); however, long-distance movements are hard to detect outside of spatially restricted study areas (Koenig et al. 1996). In this sense, all ILT banding studies have been methodologically biased against the detection of long-distance dispersal. Given this deficiency, the distances we used to define ILT population subunits should not be considered definitive, but rather a heuristic starting point for the discussion of population dynamics and to guide the design of unbiased studies of movement rates among populations. Documenting the true frequency of movement rates among ILT populations is an important topic for future study to reduce uncertainty in population models. Unbiased estimation of movement rates would require studies that dedicate significant resources to banding, recapturing, and/or resighting individuals from >1 of the “subpopulations” or “populations” described in this paper.

The distance between several ILT populations and Gulf Coast Least Tern populations is less than the distance between many interior populations (Figs. 2 and 4). If demographic connectivity is strong between interior and coastal Least Tern population, variable reproduction and immigration/emigration rates could affect both populations (Kirsch and Sidle 1999; Lott 2006). Historically, Gulf Coast Least Tern populations have not been extensively or consistently monitored. Better information about annual fluctuations in coastal Least Tern populations and demographic connectivity between the coast and interior would help to evaluate the long-term dynamics of both “populations”.

Prior descriptions of ILT distribution, including documents that have set range-wide or regional population objectives, have poorly addressed the likelihood of demographic connectivity across landscapes with near-continuous suitable habitat (USFWS 1990, 2003, 2005a). For example, ILT that breed on the Niobrara River occupy breeding habitat that is contiguous (at short dispersal distances) with breeding areas below Fort Randall and Gavins Point Dams on the Missouri River (Fig. 5). Changing conditions on all three of these river segments have clearly influenced the regional distribution of ILT over time (USFWS 2003; Lott 2006). Yet, progress toward population targets or the evaluation of management treatments for the two Missouri River segments (defined as administrative “populations” by USACE project area boundaries) are evaluated independently (USFWS 2003).

If ILT move regularly among river segments, restricting the evaluation of management actions to a single segment could be misleading. For example, positive management actions on one river segment may draw ILT away from adjacent segments, even if these are productive. If monitoring occurs only in the segment where management occurs, this movement may appear as a management-driven population increase, rather than simply the redistribution of regional breeding colonies. Conversely, if monitoring occurs only in the area that terns emigrate from to exploit new habitat on an adjacent segment, this movement may be interpreted as a local population decline rather than regional redistribution. Clearly, the frequency of movement among adjacent river segments within the same or proximate population subunits must be documented to understand the effects of local management on population size at local, regional, or larger scales. The population subunits defined here are a first step toward connecting the evaluation of progress toward population size targets or management effectiveness to biologically relevant scales.

The distribution of ILT populations is strongly tied to wide river channels where high flow events periodically redistribute large quantities of sand, increase sandbar height and extent, and set back plant succession on bare sandbars. Nearly all the rivers where ILT occur have been extensively altered. However, sandbar creation and renewal processes persist on many river segments. For example, regulation era floods and flow regimes have been very effective at creating extensive suitable nesting habitat on the Missouri River below Garrison and Gavins Point Dams (USACE 2011, 2012b); on the Lower Platte River (Sidle et al. 1993; Alexander et al. 2013); below Keystone Dam on the Arkansas River (Leslie et al. 2000; Lott and Wiley 2012a); and below Denison Dam on the Red River (Lott and Wiley 2012b). These studies have identified four different types flow regimes that lead to ILT sandbar nesting habitat renewal: (1) periodic and long-duration high dam releases that create high-elevation sandbars, which may persist for several years (the Missouri River below Gavins Point Dam, the Arkansas River below Keystone Dam, and the Red River below Denison Dam)(Fig. 7); (2) steeply falling hydrographs during the breeding season that discourage the recruitment and survival of pioneering sandbar vegetation (the Upper Red, Cimarron, Canadian, and Lower Mississippi Rivers); (3) large interannual stage differences between high nonbreeding season and low breeding season flows (e.g., the Lower Mississippi River); and (4) extreme flow variability on flashy streams, where frequent flow reversals during the growing season discourage tree recruitment or survival (e.g., the Niobrara, Lower Platte, Upper Red, Canadian, and Cimarron Rivers).

ILT population persistence (and the effectiveness of local or regional management actions) should be evaluated in the context of interactions between sandbar habitat dynamics and important ILT life history traits. For example, adult ILT have high annual survival rates and long reproductive lifespans (Renken and Smith 1995b; Akcakaya et al. 2003). Least Terns attempt to breed nearly every year after first breeding at the age of 2 or 3, and individuals have been recorded breeding at the age of 20 (Thompson et al. 1997). Given an annual adult survival rate of 0.88, the median reproductive lifespan for ILT would be ∼6 years and 25% of adults would reproduce for ∼11 years. With this many breeding attempts, periodic “boom” years, such as those that occur after major flooding events (Leslie et al. 2000; USACE 2011) may produce enough fledglings to sustain regional populations, even when many years are characterized by poor reproductive performance (Whittier 2001). Future research on ILT sandbar nesting habitat and population process should focus on the relationship between the frequency, magnitude, duration, or intensity of high flow events (Mori 2011) and the subsequent characteristics of sandbars (e.g., elevation, area, persistence) that affect ILT population dynamics at the temporal scale of the reproductive lifetime of most ILT (6–11 years).

## Conclusions

Shaffer and Stein (2000) advocated a conceptual framework to evaluate species conservation status based on the concepts of representation, resiliency, and redundancy (the “three Rs”). These concepts have been repeated, elaborated upon, and advocated as tenets of conservation planning for many years (Groves et al. 2002; Tear et al. 2005; Carroll et al. 2010; Redford et al. 2011). Representation refers to “saving populations of each species in the array of different environments in which it occurs” (Shaffer and Stein 2000). Resiliency describes the qualities “of an ecological element that allows it to persist through severe hardships” (Scott et al. 2005). Redundancy refers to having more than one population of each type so that the loss of one does not result in irreversible loss of diversity (Crandall et al. 2000).

Representation is an inherently difficult topic to address for most species, as reliable information about historical ranges is usually unavailable (e.g., the prealteration era for ILT). However, the current range of ILT includes nearly all of the sand bed Rivers of the Great Plains and Lower Mississippi Valley that occurred prior to large-scale river modification. Despite range fragmentation by reservoirs, the two main habitat types that were historically present (sandbars on large rivers and salt plains adjacent to these rivers) are still present and occupied by ILT. There are no historically known habitat types that may have been suitable for ILT that are not present and occupied today. Consequently, we believe that ILT populations are representative of their entire historic geographical, geophysical, and ecological range. This is in stark contrast to other endangered species, some of which have been delisted (e.g., Grey Wolf, *Canis lupus*; American Buffalo, *Bison bison*), that no longer occupy large portions of their former ranges (Carroll et al. 2006, 2010; Sanderson et al. 2008).

Several life history characteristics of ILT make their populations inherently resilient. In addition to high annual survival and long reproductive lifetimes (described above), the ability of ILT to renest up to three times in a single breeding season increases opportunities for successful reproduction after initial reproductive failure (Massey and Fancher 1989; Lingle 1993). ILT are generalist predators that eat a large number of native and exotic fishes across a wide range of foraging microhabitats (Tibbs 1995; Dugger 1997; Sherfy et al. 2012a; Stucker et al. 2012) and are capable of traveling long distances from colonies during foraging trips (Sherfy et al. 2012a). This dietary flexibility provides resiliency against changes in fish species composition, such as those that have occurred on some large, regulated rivers (Gido et al. 2010; Hoagstrom et al. 2011). Although there is still uncertainty regarding the frequency of long-distance dispersal for ILT, there is ample evidence of regional population redistribution to exploit high-quality nesting habitat that has appeared after large floods (Leslie et al. 2000; USACE 2011). The ability of ILT to respond rapidly to changing environmental conditions should facilitate the recolonization of former breeding areas if habitat becomes temporarily unsuitable and is later renewed after extreme flooding events (Schumm and Lichty 1963), or range expansion when flooding, habitat restoration, and other anthropogenic activities create suitable habitat in new areas (Busby et al. 1997; USACE 2011, 2012a). Finally, the nearly 3-decade persistence of small ILT populations in isolated areas that have been subject to environmental stochasticity (e.g., flooding, drought) and the continued effects of dams and water diversions suggests that even small ILT populations are resilient to multiple stressors (Castrale et al. 2004; Nelson 2004; Doster 2009).

Shaffer and Stein (2000) stressed the importance of preserving multiple functioning populations across the range of a species. This redundancy safeguards against a loss of representation if a small number of subpopulations are extirpated and ensures that negative events within a small area or region (e.g., a persistent drought) do not have catastrophic consequences for an entire population (Tear et al. 2005). This redundancy is evidenced by the large number of riverine populations of ILT, spread across >4600 km of rivers across the Great Plains and the Lower Mississippi Valley (Figs. 2, 4, 5; Table 1). The loss of a large number of these populations due to demographic or environmental stochasticity seems unlikely as many of these populations are large and have their own built in redundancy, as they comprised many colonies and individuals. The large distribution of ILT buffers local populations from permanent extirpation by increasing the likelihood of recolonization from replicate populations nearby. The large number of ILT populations in novel, anthropogenic habitat types such as navigation dike fields, dredged material disposal sites, and off-channel sites like sand and gravel mines provides additional sources for recolonization of riverine populations if these are temporarily lost.

ILT populations seem to do well against the “three-R” standard; however, ILT metapopulation dynamics remain uncertain (and the need for management remains unclear) due to the challenges of creating unbiased population projection models for Least Terns (Akcakaya et al. 2003). Inferences from previous population models have been limited by high parameter uncertainty due to chronic problems of partial observability that are difficult to overcome with Least Terns: for example, chicks are difficult to detect, most populations violate closure assumptions at the temporal scale of a breeding season, renesting is often impossible to account for, and survival estimates are uncertain due to the problem of separating death from permanent emigration (Smith and Renken 1993; Thompson et al. 2000; Kress and Hall 2002; Akcakaya et al. 2003). This type of demographic parameter uncertainty is compounded by the absence of robust studies of movement rates among population subunits and uncertainty related to interactions between postregulation flow regimes and the foundational processes of habitat formation and loss. Overcoming these sources of uncertainty will be challenging, particularly given the high costs of conducting large-scale studies of movement rates or habitat dynamics on sand bed rivers that are spread across thousands of km that are often difficult to access by boat. We recommend constructing habitat-based metapopulation models at the scale of the listed ILT entity, linked with Least Tern populations on the Gulf Coast. Given the uncertainty that will accompany model inputs, sensitivity analyses will be useful to suggest inferential limits for ILT population models and future research efforts with the highest likelihood of reducing uncertainty.
